# mTOR deletion ameliorates CD4 + T cell apoptosis during sepsis by improving autophagosome-lysosome fusion

**DOI:** 10.1007/s10495-022-01719-y

**Published:** 2022-04-18

**Authors:** Hao Wang, Guangxu Bai, Jianwei Chen, Wen Han, Ran Guo, Na Cui

**Affiliations:** 1 Department of Critical Care Medicine, Beijing Jishuitan Hospital, 100035 Beijing, China; 2 Department of Critical Care Medicine, State Key Laboratory of Complex Severe and Rare Diseases, Peking Union Medical College Hospital, Chinese Academy of Medical Science and Peking Union Medical College, 100730 Beijing, China

**Keywords:** mTOR, Sepsis, Autophagosome-lysosome fusion

## Abstract

Autophagy dysfunction contributes to CD4 + T cell apoptosis during sepsis leading to impairment of adaptive immunity. However, the underlying mechanism is unclear. The mammalian target of rapamycin (mTOR) pathway modulates CD4 + T cell survival during sepsis through mechanisms that are not fully understood. We developed a mouse model of sepsis through cecal ligation and puncture (CLP) to investigate dynamic changes in autophagy in CD4 + T cells. We used T cell specific-mTOR/tuberous sclerosis complex 1 (TSC1)-knockout mice to explore the roles of the mTOR pathway in modulating autophagy during sepsis. We observed reduced fusion of autophagosomes with lysosomes in the CD4 + T cells of CLP mice, which may represent a characteristic feature of autophagy dysfunction. Deletion of mTOR relieved autophagosome-lysosome fusion dysfunction and ameliorated apoptosis of CD4 + T cells in CLP mice, but this rescued phenotype was abolished by treatment with bafilomycin A1, a specific A-L fusion inhibitor. We further explored the underlying molecular mechanism and found that phosphorylation levels of transcription factor EB were significant higher in CLP mice and that expression of A-L fusion protein SNAREs were restricted, both of which were ameliorated by mTOR deletion. Taken together, these results suggest that the mTOR pathway plays a critical role in regulation of CD4 + T-cell apoptosis during sepsis, partly through regulation of A-L fusion-related protein transcription.

## Introduction

Sepsis is a form of life-threatening organ dysfunction that results from exaggerated host immune responses to disseminated infection [1]. Although guidelines and cluster therapies are constantly being updated, the morbidity and mortality of sepsis are still high, making it an important global health issue. According to a study published in 2020, sepsis affects an estimated 50 million individuals worldwide each year and accounts for nearly 20% of global deaths [2]. New treatments for sepsis are urgently needed. Sepsis is characterized by simultaneous pro-inflammatory and anti-inflammatory reactions, resulting in an exaggerated inflammatory response followed quickly by immunoparalysis [3]. Adaptive immune responses mediated by CD4 + T cells play important roles in defense against infection, but are affected during the early stages of sepsis. Losses of CD4 + T cells via apoptosis occurs following sepsis onset and plays an important role in progression of immune suppression, which is closely associated with poor outcomes [4–6]. Therefore, we designed the present study to investigate the mechanisms underlying excessive apoptosis of CD4 + T cells during sepsis. The results of our study may help in developing novel therapeutic strategies to improve outcomes in septic patients.

The mammalian target of rapamycin (mTOR) signaling pathway is an evolutionarily conserved mechanism that primarily controls cell metabolism and survival [7]. The pathway consists of two protein complexes: mTOR complex (mTORC)1 and mTORC2 [8]. mTORC1 plays an important role in cell death regulation (e.g., autophagy and apoptosis). mTORC1 functions as a promotor of protein synthesis mainly though phosphorylation of downstream molecules such as S6 kinase (S6K). Tuberous sclerosis complex 1 (TSC1) negatively regulates mTOR activity [9]. The mTOR pathway influences CD4 + T cell survival during sepsis [10, 11]. However, the molecular and cellular mechanisms underlying regulation of CD4 + T cell survival remain incompletely understood.

Autophagy is a homeostasis-maintaining system that plays a protective role during sepsis by preventing various cells from undergoing apoptosis (e.g., heart, liver, lung, and kidney cells) [12–15]. Recent work has indicated the vital role of autophagy in lymphocyte survival [5]. The complete autophagy process includes phagophore formation, autophagosome maturation, autophagosome-lysosome fusion, and degradation. This process is defined as autophagic flux, and complete autophagic flux ensures the proper recycling of damaged organelles and large molecules in cells, which is essential for maintenance of homeostasis under stress [16]. Previous studies indicated a relationship between autophagy disorders and exaggerated CD4 + T cell apoptosis during sepsis. However, the major focus of prior studies was the formation of autophagosomes, and comparatively little is known regarding the other steps in autophagy flux [17]. Classical studies by Franco Fortunato et al. found that autophagosome-lysosome fusion (A-L fusion) is attenuated in a variety of cells during endotoxin-induced systemic disorders, exacerbating cell death and tissue injury [18]. We hypothesized that impaired autophagosome degradation may result from inhibited fusion with lysosomes. Therefore, we designed the present study to investigate the molecular mechanisms underlying autophagic flux deficiency in CD4 + T cells during sepsis and to explore the roles of mTOR pathway in modulating CD4 + T cell survival.

## Materials and methods

### Mice

mTOR^loxp/loxp^, TSC1^loxp/loxp^ and Lck-Cre mice were kindly provided by Dr. Yong Zhao (State Key Laboratory of Biomembrane and Membrane Biotechnology, Institute of Zoology, Chinese Academy of Sciences, Beijing, China). We crossed TSC1^loxp/loxp^ and mTOR^loxp/loxp^ mice with Lck-Cre mice (Cre expression under the control of lymphocyte-specific protein tyrosine kinase, Lck) to obtain the F1 generation Lck-Cre:mTOR^loxp/−^ mice and Lck-Cre:TSC1^loxp/−^ mice, respectively. We interbred F1 generation mice in each group and obtained F2 generation Lck-Cre: mTOR^loxp/loxp^ and Lck-Cre;TSC1^loxp/loxp^ mice. Littermates lacking Lck-Cre served as controls. All animals were maintained in the Animal Laboratory of Peking Union Medical College Hospital under specific pathogen-free conditions. Experiments were performed in accordance with the National Institutes of Health Guidelines for the Use of Laboratory Animals.

### Mouse sepsis model

A cecal ligation and puncture (CLP) mouse model of sepsis was established based on the protocol of Rittirsch et al. [19]. Briefly, mice were anesthetized via isoflurane inhalation, then the abdomen was opened surgically and the cecum was ligated halfway between the distal pole and the base. The cecal stump was punctured once with a 22-gauge needle and a small amount of stool was extruded. The cecum was placed back into its normal intraabdominal position and the abdomen was closed. Next, 1 mL of saline was administered subcutaneously to each animal for fluid resuscitation. Animals in the sham group underwent the same surgical procedure without ligation or puncture. Bafilomycin A1 (1 mg/kg; Solarbio, Beijing, China) was administered intraperitoneally 1 h after the CLP operation. Rapamycin (6 mg/kg; Shanghai yuanye Bio-Technology, Shanghai, China) was administered intraperitoneally 3 h after the CLP operation. Mice were returned to cages and provided with food and water. The mortality rate was monitored every hour until 24 h after surgery.

### Lymphocyte isolation and cell sorting

Mice were euthanized 12 h after surgery and spleens were harvested. Spleens were pressed gently with glass slides. The resulting homogenates were washed with phosphate-buffered saline and ammonium-chloride-potassium lysis buffer was used to lyse red blood cells. Centrifuged splenocytes were resuspended in RPMI-1640 medium (Sigma-Aldrich, St. Louis, MO, USA). Splenocytes were stained with biotin-conjugated anti-CD4 antibody on ice for 30 min, washed, incubated with magnetic streptavidin beads for 15 min, and resuspended in cell-sorting buffer. CD4 + T cells were isolated by negative selection using separate columns. The purity of the resulting CD4 + T cells was determined as > 90%. The sorted cells were subsequently used for analysis of apoptosis, western blotting, quantitative real-time polymerase chain reaction (qPCR), and transmission electron microscopy (TEM).

### Transmission electron microscopy (TEM)

Purified CD4 + T cells were fixed with 4% paraformaldehyde and 2.5% glutaraldehyde in sodium phosphate buffer (0.1 M, pH 7.2) overnight at 4 °C. Cells were post-fixed with 1% OsO_4_ in distilled water for 1 h at room temperature prior to TEM observation. Lymphocytes were embedded in 2% agar prior to dehydration via a graded ethanol series. The samples were embedded in Epon 812. Ultrathin sections were cut using an ultramicrotome (Ultracut E) and aqueous uranyl acetate and lead citrate were used for staining. A transmission electron microscope (JEM1230; Jeol, Tokyo, Japan) was used for observation. Autophagosomes were defined as double-membraned structures that contain cytoplasm with damaged organelles. Autolysosomes were defined as single-membraned vesicles with cytoplasmic or organellar debris in various stages of degradation.

### Apoptosis assay

An Annexin V/fluorescein isothiocyanate (FITC) apoptosis detection kit (BD Biosciences, San Diego, CA, USA) was used for assessment of apoptosis. After staining, CD4 + T cells were examined using a FACSCalibur instrument (BD Biosciences) and Cell Quest Pro software. The excitation wavelength was 488 nm and the emission wavelength was 525 nm. A minimum of 10,000 cells were analyzed per sample and dot plots were prepared using Flowjo software.

### Western blotting

Radioimmunoprecipitation assay buffer was used for protein extraction. Tissue suspensions were centrifuged for 15 min at 4 °C, 14,000 × g, and the upper supernatant was collected. Total protein concentration was measured using the bicinchoninic acid method. An equal amount of protein (30 µg) for each sample was subjected to SDS-PAGE and transferred to polyvinylidene difluoride membranes. The membranes were blocked with 5% nonfat milk and incubated with specific primary antibodies overnight. The membranes were washed three times with Tris-buffered saline containing 0.1% Tween-20, incubated with a corresponding horseradish-peroxidase-conjugated secondary antibody for 1 h, and then developed on X-ray films using chemiluminescent reagents. Images were captured using a Bio-Rad ChemiDoc XRS + instrument (Bio-Rad, Hercules, CA, USA) and densitometric analyses were performed using QuantityOne software (Bio-Rad). The antibodies used for western blotting were as follows (all from Affinity BioSciences, Cincinnati, OH, USA): phospho-mTOR (Ser2448) antibody, phospho-p70 S6K (Thr389/Thr412) antibody, sequestome 1 (SQSTM1)/p62 antibody, LC3A/B antibody, syntaxin 17 (STX17) antibody, vesicle associated membrane protein 8 (VAMP8) antibody, synaptosomal-associated protein 29 (SNAP29) antibody, phospho-transcription factor EB (TFEB) (Ser211) antibody, and beta-actin antibody.

### Quantitative polymerase chain reaction (qPCR)

Total RNA was extracted from sorted CD4 + T cells using TRIzol Reagent (Tiangen Biotech, Beijing, China). The IQ5 detection system and SYBR Green Real time PCR Master Mix were used for qPCR analysis. The following PCR primers were used: STX17, 5’-GCTTGAGCCAGCGATACAGA-3’ and 5‘-TATTGGAGCGCAGTTGCTGA- 3’; VAMP8, 5’-GGACCACCTCCGAAACAAGA-3’ and 5‘-AGGGCTCCTCTTGGCACATA- 3’; and SNAP29, 5’-AGCCCAACAGCAGATTGAAA-3’ and 5‘-AAAACTCAGCAGAACAGCTCAA-3’. β-actin was used as an internal control and fold changes were calculated by relative quantification (2^−∆∆Ct^). Each experiment was conducted three times.

### Statistical analysis

Data were analyzed using IBM SPSS software version 19.0 (IBM, Armonk, NY, USA). Data for normally distributed continuous variables were presented as means ± standard deviations (SDs). Differences were assessed using analysis of variance followed by the least significant difference test. Values of P < 0.05 were considered statistically significant.

## Results

### Incomplete autophagic flux occurred in the CD4 + T cells of CLP mice, including impaired autophagosome-lysosome fusion

**Fig. 1:**
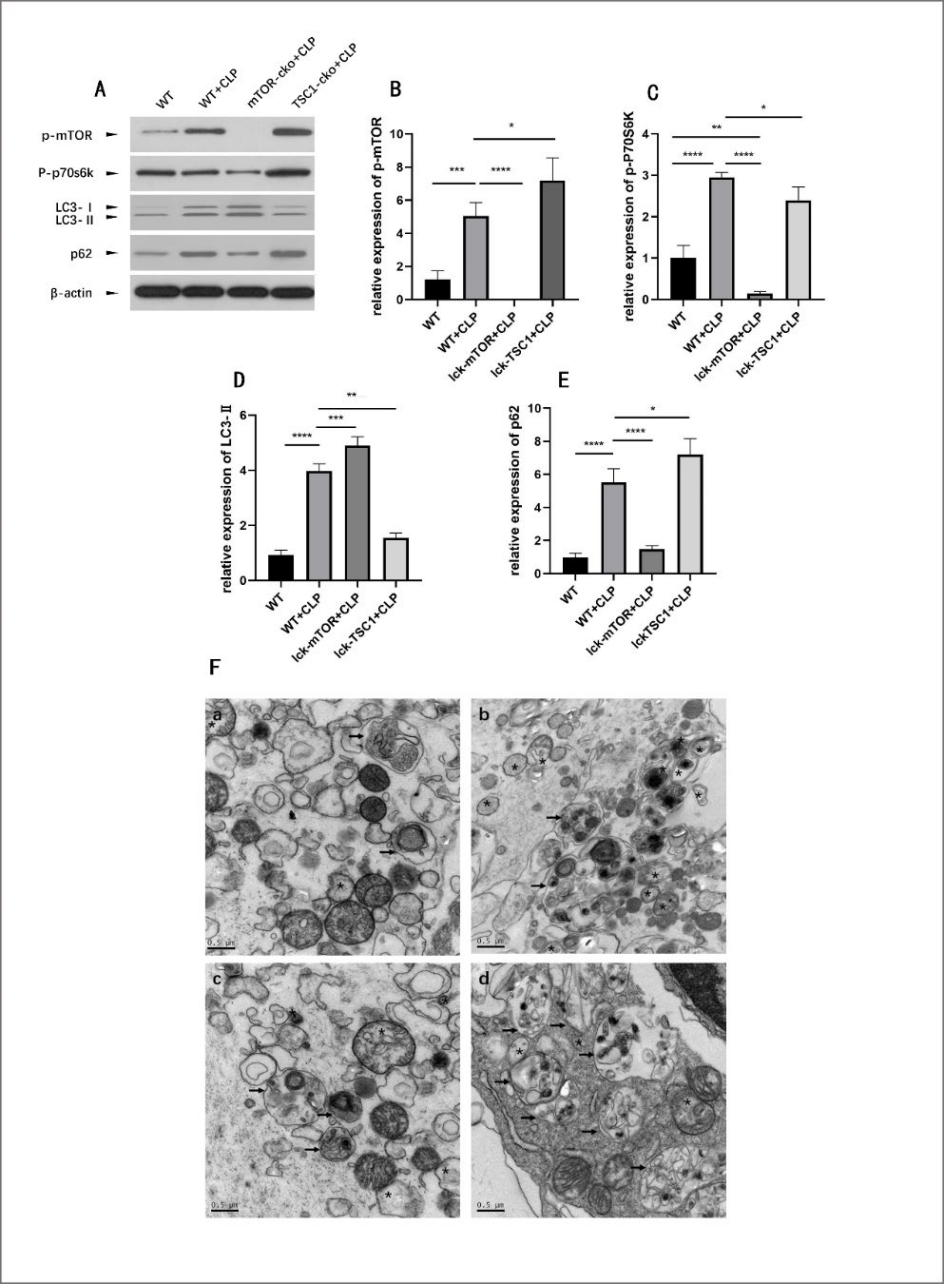
Mammalian target of rapamycin (mTOR) activity and autophagic flux in CD4 + T cells. (A–E) Protein levels of phospho (p)-mTOR, p-P70 S6 kinase (S6K), LC3II/I and p62/sequestome 1 (SQSTM1) in CD4 + T cells were quantified by western blotting in wild-type (WT) mice, mTOR knockout via Cre expression under the control of lymphocyte-specific protein tyrosine kinase (Lck) (Lck-mTOR) cecal ligation and puncture (CLP) mice, and tuberous sclerosis complex 1 (TSC1) knockout (Lck-TSC1) + CLP mice. The amount of each protein level was normalized to that of β-actin. Means ± standard deviations (SDs) of four mice per group are shown. **P < 0.01, ***P < 0.001, ****P < 0.0001. (F) Ultrastructural features of CD4 + T cells were investigated using transmission electron microscopy (TEM). Autophagosomes were double-membrane vacuoles containing cytosol or organelles (asterisk). Autolysosomes were single-membrane structures containing digested cytoplasmic components (black arrow). (Fa) In control mice, CD4 + T cells had normal morphologies, revealing baseline autophagy status. (Fb) WT + CLP mice displayed increased autophagic vacuolization but no significant increase in autolysosome frequency. Large autolysosomes containing abundant contents were seen. (Fc) Lck-mTOR + CLP mice showed more autophagic vacuolization and more autolysosomes. (Fd) Autophagosomes and autolysosomes were fewer in Lck-TSC1 + CLP mice

To characterize autophagy levels of CD4 + T cells in a mouse sepsis model, we assessed the expression of the autophagy proteins LC3II/I and p62/SQSTM1 in CLP mice and control mice. LC3 is an autophagy-related protein (Atg) 8-family protein and mainly functions at the stage of phagophore expansion. LC3 consists of two isoforms, LC3I and LC3II, and the ratio of LC3II/I is widely considered to reflect dynamic changes in autophagy flux [20]. The p62/SQSTM1 protein is an autophagosome substrate and functions as a selective autophagy receptor for degradation of ubiquitinated substrates. The level of p62/SQSTM1 is generally considered to be inversely correlated with autophagic activity [21]. In mice with sepsis, LC3II/I expression in CD4 + T cells was significantly higher compared with that of control mice (Fig. 1D). Levels of p62/SQSTM1, which is a marker protein of incomplete autophagic flux, were also higher in septic mice (Fig. 1E). These results indicated incomplete autophagic flux and impaired autolysosome degradation in the CD4 + T cells of septic mice. We further used TEM to observe changes in ultrastructural features during autophagic flux in CD4 + T cells. The numbers of autophagosomes in the CD4 + T cells of septic mice were significantly higher than those of control mice, and the size of autophagosomes was larger (Fig. 1 F). These results indicated that in CD4 + T cells of septic mice, autophagosomes cannot fuse normally with lysosomes to form autolysosomes.

### mTOR pathway activity was elevated in CLP mice

To investigate the activity of mTOR signaling in septic mice, we assessed levels of phosphorylated mTOR and p70 S6K, and found that both were elevated in septic mice (Fig. 1B–C). These results indicated that the mTOR pathway was hyperactivated in septic mice, and may play a central role in the regulation of CD4 + T cell apoptosis during sepsis.

### mTOR knockout in CD4 + T cells ameliorated A-L fusion disorder in septic mice

To further investigate the mechanisms of mTOR pathway regulation of CD4 + T cells during sepsis, two types of knockout mice were used: T-cell-specific mTOR knockout (Lck-mTOR) mice, and T-cell-specific TSC1 knockout (Lck-TSC1) mice. In Lck-mTOR + CLP mice, expression of LC3 II/I was significantly higher and that of p62/SQSTM1 was lower in CD4 + T cells compared with wild-type (WT) CLP mice. This finding indicated that autophagic flux disorder induced during sepsis was relieved by mTOR knockout. In TSC1-cko + CLP mice, levels of LC3 II/I and p62/SQSTM1 were both lower compared with WT mice (Fig. 1D–E). This finding indicated that hyperactivation of the mTOR pathway may restrain the autophagy process including the initiation stage and autophagosome-lysosome fusion.

### mTOR knockout reduced CD4 + T cell apoptosis in septic mice, and reduction of apoptosis could be reversed using bafilomycin A1

**Fig. 2:**
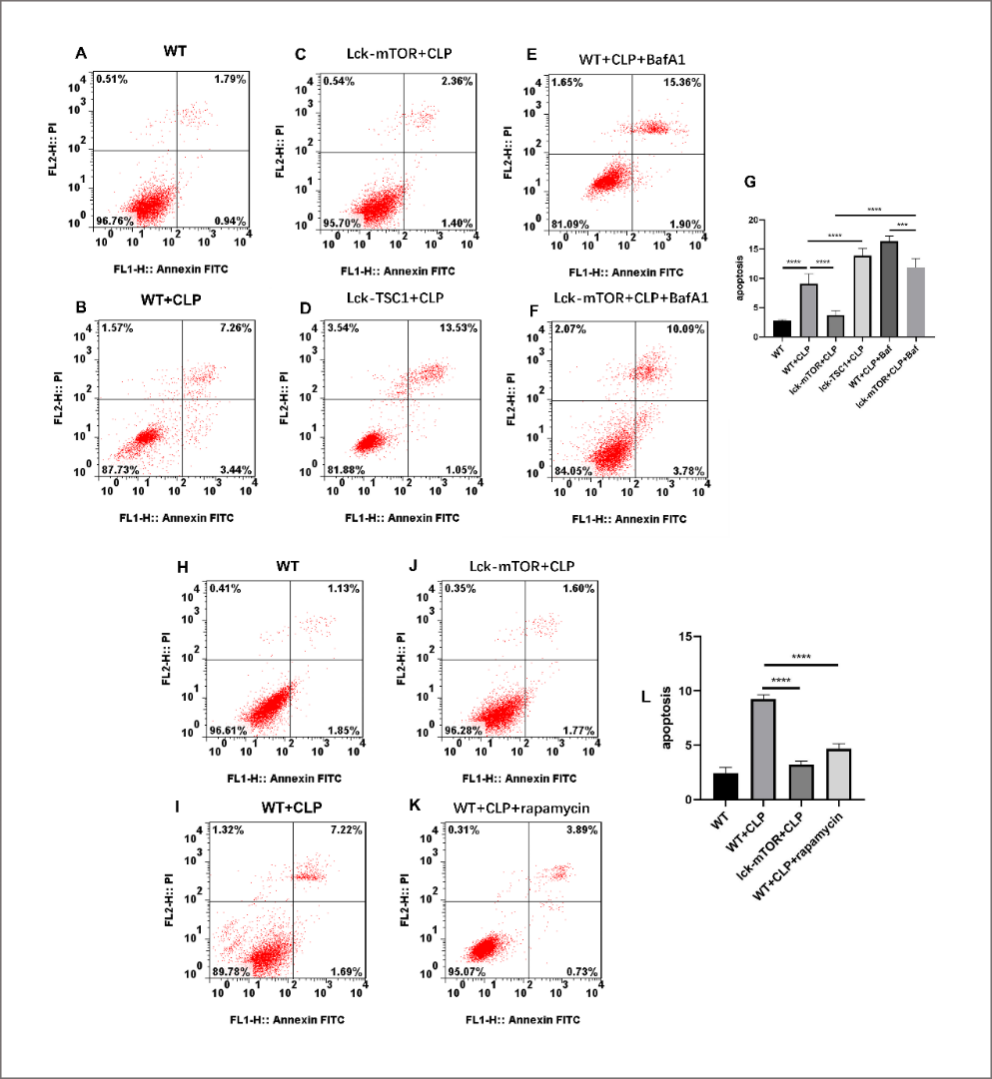
Evaluation of apoptosis of CD4 + T cells during sepsis. (A–F, H–K) The subpopulation of Annexin V-positive and propidium iodide (PI)-negative CD4 + T cells was considered to be undergoing early apoptosis, while the subpopulation of Annexin V-positive and PI-positive CD4 + T cells was considered to be undergoing late apoptosis. (G, L) Apoptosis rate of CD4 + T cells as measured via the ratio of Annexin V-positive and PI-positive/negative CD4 + T cells. Means ± standard deviations (SDs) of four mice per group are shown. **P < 0.01, ∗∗∗∗p < 0.0001

We further investigated CD4 + T cell survival during sepsis. We assessed apoptosis of CD4 + T cells using flow cytometry to measure incorporation of Annexin V-FITC/propidium iodide (PI). Compared with control mice, the percentages of Annexin V + and PI- CD4 + T cells in septic mice were significantly elevated. In Lck-mTOR + CLP mice, the apoptosis rate of CD4 + T cells was reduced compared with WT + CLP mice, while the apoptosis rate was elevated in Lck-TSC1 mice (Fig. 2 A–D). These results indicated that inhibition of the mTOR pathway decreased apoptosis of CD4 + T cells in septic mice. We used bafilomycin A1 to specifically block fusion of autophagosomes and lysosomes. In Lck-mTOR + CLP mice treated with bafilomycin A1, the apoptosis rate of CD4 + T cells was higher compared with mice that did not receive bafilomycin A1 (Fig. 2 F) and lower compared with mice in WT + CLP + bafilomycin A1 group (Fig. 2E). This result indicated that the protective effects of mTOR deletion against CD4 + T cell apoptosis were attenuated by bafilomycin A1.

We used rapamycin, a specific blocker of mTOR, to confirm the result obtained by genetic approach. In WT + CLP mice treated with rapamycin, CD4 + T cell apoptosis rate was significantly reduced, which is consistent with the protective effect of mTOR knockout (Fig. 2 H-K).

### mTOR knockout reduced phosphorylation of TFEB and restored SNAREs transcription in septic mice

**Fig. 3:**
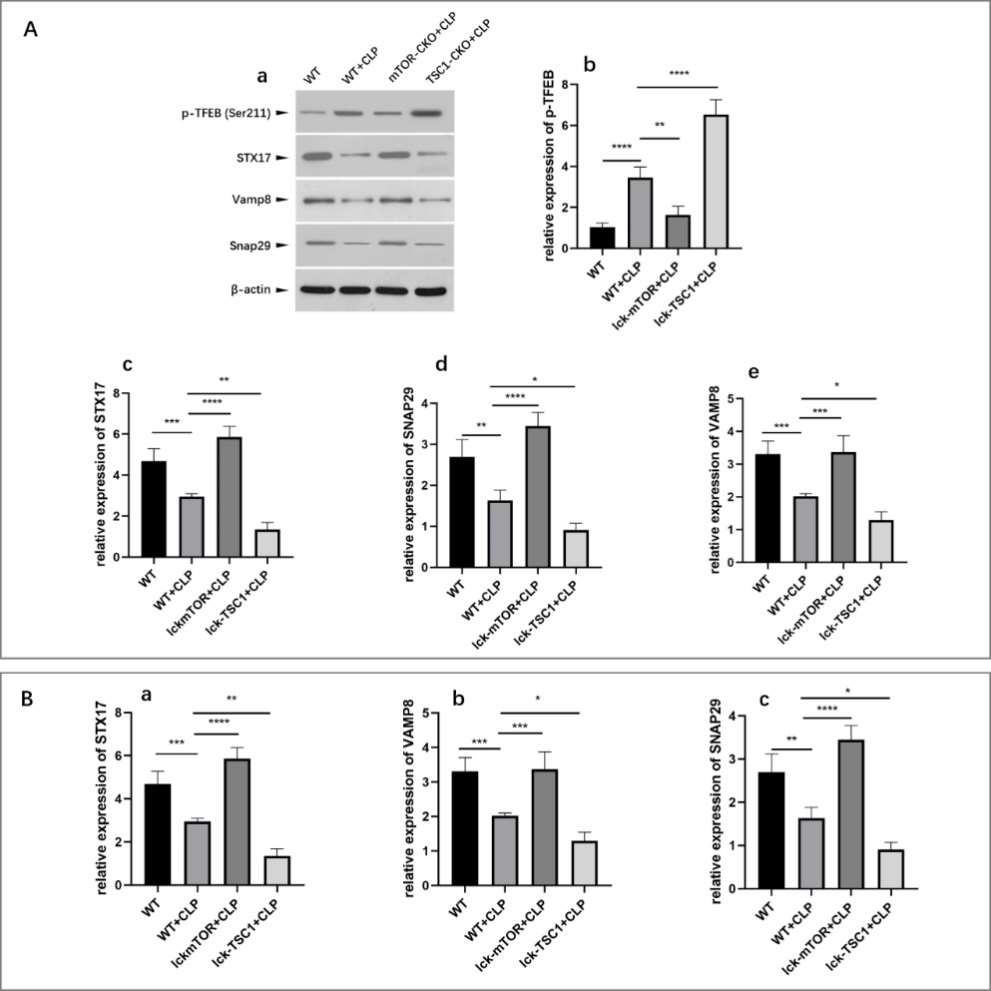
Transcription factor EB (TFEB) phosphorylation and SNARE expression in septic mice. (A, a–e) Protein levels of phospho (p)-TFEB, syntaxin 17 (STX17), vesicle associated membrane protein 8 (VAMP8), and synaptosomal-associated protein 29 (SNAP29) in CD4 + T cells were quantified by western blotting in wild-type (WT) sham-treated mice, WT CLP mice, mammalian target of rapamycin (mTOR) knockout via Cre expression under the control of lymphocyte-specific protein tyrosine kinase (Lck) (Lck-mTOR) CLP mice, and tuberous sclerosis complex 1 (TSC1) knockout (Lck-TSC1) CLP mice. The amount of each protein level was normalized to that of β-actin. Means ± standard deviations (SDs) of four mice per group are shown. *P < 0.05, **P < 0.01, ***P < 0.001, ****P < 0.0001

To investigate the molecular mechanisms underlying autophagosome-lysosome fusion disorders in the CD4 + T cells of septic mice, we measured the expression of STX17, VAMP8, and SNAP29. In the CD4 + T cells of septic mice, expression of STX17, VAMP8, and SNAP29 was significantly lower compared with control mice (Fig. 3 A, c–e). We further assessed the mRNA abundance of STX17, VAMP8, and SNAP29 transcripts by qPCR. The mRNA levels of these three molecules were significantly lower in septic mice compared with control mice, indicating reduced expression of these genes at the transcription level (Fig. 3B, a–c). Next, we measured the phosphorylation level of TFEB, a master regulator of SNARE transcription. We found that phosphorylation of TFEB was significantly elevated in CLP mice compared with control mice (Fig. 3 A, b). These results indicated that sepsis-induced autophagosome-lysosome fusion disorders may be the consequence of impaired TFEB activity, resulting in downregulation of the transcription of SNARE components.

To explore the effects of targeting the mTOR pathway on A-L fusion, we analyzed TFEB phosphorylation and assessed the expression of SNARE components at both the transcriptional and translational levels in knockout mice. In Lck-mTOR + CLP mice, we observed reduced phosphorylation of TFEB compared with WT + CLP mice (Fig. 3 A, b) accompanied by elevated expression of SNARE components (Fig. 3 A, c–e; Fig. 3B, a–c). These results indicated that mTOR may function as a suppressor of SNARE expression by enhancing TFEB phosphorylation.

## Discussion

Sepsis is characterized by simultaneous pro-inflammatory and anti-inflammatory reactions, resulting in both exaggerated inflammatory responses followed quickly by immunoparalysis. Dysregulation of CD4 + T cell apoptosis contributes to immunosuppression, which is universally observed in sepsis [22]. Apoptosis is known as “type 1 programmed cell death,” and autophagy as “type 2 programmed cell death”. Crosstalk between the two types of cell death has been previously described. Autophagy is regarded as a protective mechanism from apoptosis in CD4 + T cells during sepsis, and deficient autophagy accelerates apoptosis [23]. Oami and colleges reported inhibition of autophagy processes of CD4 + T cells during sepsis, which were proposed to lead to exaggerated apoptosis and immunosuppression [24]. Our previous study also found increased levels of LC3II/I and p62 in the CD4 + T cells of septic mice, indicating insufficiency of autophagy; this finding is consistent with the results of Oami et al. However, the mechanism underlying autophagy insufficiency remained unclear. Autophagy is a highly conserved catabolic process whereby selected cellular components are delivered to and degraded within lysosomes to maintain cellular homeostasis. Complete autophagic flux includes phagophore formation, autophagosome maturation, autophagosome-lysosome fusion, and degradation, and is essential for maintenance of homeostasis under stress [16]. Therefore, in the present study, we constructed a CLP mouse model of sepsis to explore the dynamics of autophagic flux in CD4 + T cells during sepsis. We used TEM to directly observe autophagic flux and observed increased numbers and sizes of autophagosomes in septic mice. These results demonstrated incomplete autophagic flux in the CD4 + T cells of mice during the early stages of sepsis, with manifestations including impaired autophagosome-lysosome fusion.

We further investigated potential pathways mediating A-L fusion dysfunction in CD4 + T cells during sepsis. We found that mTOR phosphorylation levels were elevated in septic mice compared with those in control mice. The mTOR pathway is extensively involved in lymphocyte biology, including roles in lymphocyte development, activation, and differentiation. mTOR is a negative regulator of autophagy. During stress, mTOR is inactivated and dissociates from dephosphorylated Unc-51 like autophagy activating kinase 1 (ULK1), which phosphorylates and forms a complex with ATG13 and RB1 inducible coiled-coil 1 (RB1CC1)/ FAK family kinase-interacting protein of 200 kDa (FIP200) to initiate nucleation of the phagophore. mTOR also negatively regulates fusion and degradation of autophagosomes in cardiomyocytes during ischemia/reperfusion injury [20]. On the basis of these results, we hypothesized that the mTOR pathway may also play a vital role in A-L fusion failure in CD4 + T cells during sepsis. We used mTOR/TSC1 knockout mice to test our hypothesis. We found that CLP mice with mTOR deletions had significantly higher expression of LC3II/I and lower expression of p62 compared with WT + CLP mice, suggesting that autophagic flux was ameliorated. In CLP mice with TSC1 deletions, expression of both LC3II/I and p62 was decreased compared with WT + CLP mice, reflecting less autophagy initiation and an overall decline in autophagic flux. As shown by TEM, the CD4 + T cells of CLP mice with mTOR deletions had significantly more autophagosomes compared with those of WT + CLP mice. By contrast, in TSC1-deleted CLP mice, the numbers of autophagosomes and autolysosomes were both decreased. These results demonstrated that the mTOR pathway was involved in regulation of A-L fusion in CD4 + T-cells during sepsis.

Autophagy plays a protective role in the survival of lymphocytes during sepsis. Figure 2 shows anabatic CD4 + T cell apoptosis in CLP mice compared with control mice. In CLP mice with mTOR gene knockouts, CD4 + T cell apoptosis was mitigated. We hypothesized that the anti-apoptotic effect of mTOR deletion may be a consequence of relieved A-L fusion dysfunction. We used bafilomycin A1, a specific inhibitor of A-L fusion, to test our hypothesis. In Lck-mTOR + CLP mice treated with bafilomycin A1, apoptosis of CD4 + T cells was significantly higher compared with Lck-mTOR + CLP mice treated with saline. These results suggested that A-L fusion modulation was an important factor linking the mTOR pathway with improved CD4 + T cell survival.

We used a chemical approach to consolidate our conclusion. WT mice were treated with rapamycin 3 h after CLP operation and flowcytometry was performed to measure the apoptosis rate. The results revealed a significantly reduced apoptosis in CD4 + T cells in WT + CLP + rapamycin mice. This indicated a possibility of medical treatment in modulating CD4 + T cells apoptosis during sepsis through pharmacotherapy.

A major event during autophagosome-lysosome fusion is the SNARE complex-mediated fusion process. According to their localization, SNAREs can be divided into four motifs: Qa, Qb, Qc, and R. The Q-SNAREs including STX17 and SNAP29 form a Qabc bundle on autophagosome membranes that subsequently forms a complex with R-SNAREs (e.g., VAMP8) on lysosomes, mediating their fusion [25]. TFEB is a member of the microphthalmia (MiT/TFE) helix–loop–helix leucine-zipper (bHLH-Zip) family of transcription factors and it is recognized as a master modulator of autophagy [26]. Transcription of SNARE motifs is under the control of TFEB [16]. TFEB is under regulatory control of mTOR via phosphorylation, which determines the localization of TFEB. When phosphorylated by mTOR at S142 and S211, TFEB is mainly cytosolic and inactive [27]. We hypothesized that TFEB and SNAREs may play key roles in the modulation of A-L fusion by mTOR during sepsis, a possibility that has not been previously investigated.

In septic mice, expression levels of STX17, VAMP8, and SNAP29 proteins in CD4 + T cells were lower compared with control mice, and qPCR analyses of transcript abundance showed the same trends. We also observed decreased levels of TFEB and increased levels of phosphorylated TFEB in septic mice. We used knockout mice to investigate whether phosphorylation of TFEB and expression of SNAREs was associated with mTOR activity. In CLP mice with mTOR deletions, total TFEB and phosphorylated TFEB levels were both elevated, indicating that TFEB activity was enhanced. Expression of STX17, VAMP8, and SNAP29 was restored at both the protein and mRNA levels in CLP mice with mTOR deletions compared with CLP + WT mice. Taken together, these findings indicated that TFEB and SNARE may mediate part of the function of the mTOR pathway in CD4 + T-cell A-L fusion.

## Conclusions

**Fig. 4:**
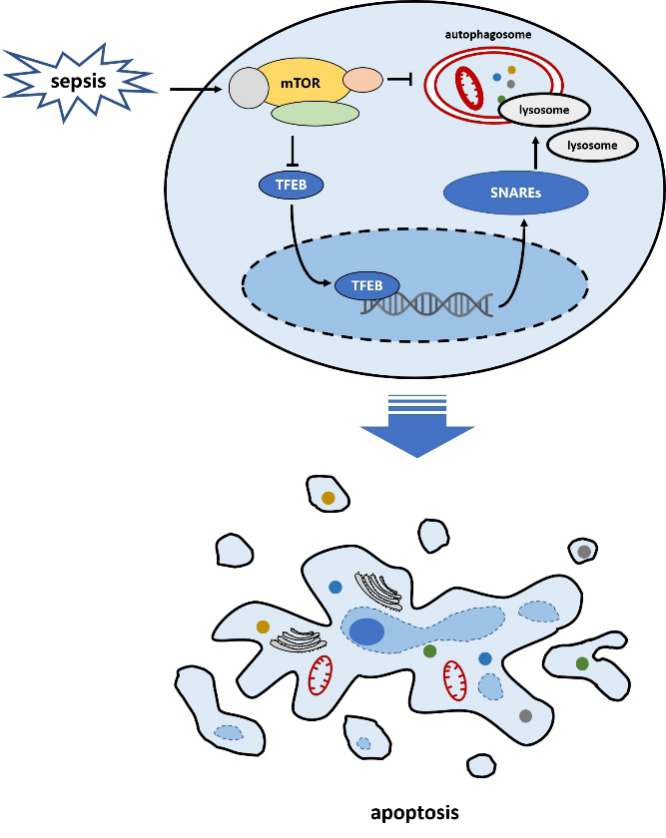
Sepsis-induced A-L fusion dysfunction exacerbates CD4 + T cell apoptosis. Mammalian target of rapamycin (mTOR) may regulate the expression of A-L fusion-related proteins including SNAREs through phosphorylation of transcription factor EB (TFEB)

As shown in Fig. 4, our data suggest that A-L fusion dysfunction is an important mechanism mediating CD4 + T cell apoptosis in sepsis. Our results also revealed the role of mTOR in regulating A-L fusion in CD4 + T cells during sepsis. The ability of mTOR to impact this important cellular process may help to reveal novel therapeutic targets in sepsis.

## Data Availability

The data that support the findings of this study are available from the corresponding author upon reasonable request.
